# A Modeling Framework for System Restoration from Cascading Failures

**DOI:** 10.1371/journal.pone.0112363

**Published:** 2014-12-04

**Authors:** Chaoran Liu, Daqing Li, Enrico Zio, Rui Kang

**Affiliations:** 1 School of Reliability and Systems Engineering, Beihang University, Beijing, China; 2 Science and Technology on Reliability and Environmental Engineering Laboratory, Beijing, China; 3 Chair on Systems Science and the Energetic challenge, European Foundation for New Energy-Electricite’ de France, Ecole Centrale Paris and Supelec, Paris, France; 4 Dipartimento di Energia, Politecnico di Milano, Milano, Italy; Center of nonlinear, China

## Abstract

System restoration from cascading failures is an integral part of the overall defense against catastrophic breakdown in networked critical infrastructures. From the outbreak of cascading failures to the system complete breakdown, actions can be taken to prevent failure propagation through the entire network. While most analysis efforts have been carried out before or after cascading failures, restoration during cascading failures has been rarely studied. In this paper, we present a modeling framework to investigate the effects of in-process restoration, which depends strongly on the timing and strength of the restoration actions. Furthermore, in the model we also consider additional disturbances to the system due to restoration actions themselves. We demonstrate that the effect of restoration is also influenced by the combination of system loading level and restoration disturbance. Our modeling framework will help to provide insights on practical restoration from cascading failures and guide improvements of reliability and resilience of actual network systems.

## Introduction

Cascading failure is a common mechanism of large-scale failures in complex network systems, such as electric power transmission grids, water/gas delivery systems, railways, etc. [1]–[7]. For a practical example, we can refer to the large-scale blackouts of electric power transmission systems resulting from cascading failures initiated by component overloads [6], [8]. Occurrences of cascading failures are found statistically more significant than that expected by theory [6], [9]. Given the vital societal importance of these critical infrastructures, there is a strong interest in the studies for the design, implementation and evaluation of effective restoration strategies against cascading failures, which rescue systems from the brink of collapse and avoid the amplification of their consequences [10]–[13].

Efforts have been carried out to study how to reduce the frequency, duration, intensity and extent of cascading failures. There are many design measures to avoid cascading failures, such as robust structures [14]–[19], capacity and structural redundancy design [19]–[22] and n-1 criterion [23], by which cascading failures can hardly be eliminated [24]. After the failure cascades, black-start [25], [26], system reconfiguration [27], [28] and corrective restoration [29], [30] are used to bring the system back to its normal operation conditions.

While complete prevention against cascading failures in design stage proves impossible and post-actions only passively recover systems at a large cost, active in-process restoration can mitigate cascading failure during its evolution, leading the system to a stable state. The primary objective of restoration during the process of cascading failures is to take actions to prevent failures from unfolding to catastrophic failures and eventually to minimize the damage, e.g. minimizing the unserved loads in an electric power transmission grid. For example, references [31], [32] propose three different strategies based on line switching to minimize the consequences of cascading failures on the entire system, on predetermined areas of the system or on both within a multi-objective optimization framework. References [33]–[36] introduce and analyze some restoration planning and restoration actions. Based on the development of fast recovery technology [37], it is possible to mitigate and rescue the system from the cascading failures through real time restoration of network components. Going back to the example of the electric power transmission grid, restoration against cascading failures may be achieved in practice through real-time controlled islanding [38], [39], selective load shedding [38], [39], wide area monitoring [40], real-time fault analysis and validating relay operations [41], etc.

In this paper, we present a novel modeling framework for analyzing restoration in network systems subject to cascading failures. The framework is used to study the effects of different restoration strategies in terms of restoration timing and strength: *t_r_*, the restoration timing in the process of the cascading failure, and *p_r_*, the restoration strength, which is quantified by the probability of repairing a failed component. Repair here means full, immediate recovery which can be realized in practice by utilizing fast recovery technology. We study how different restoration strategies described in terms of the two basic quantities (*t_r_* and *p_r_*) influence the overall system reliability.

## Description of the Restoration Model

We first consider an unweighted and fully connected network of *N* identical components [42]. The loading-dependent model proposed in [43] is adopted to describe the dynamics of cascading failures. The model is analytically tractable and captures some essential features of the cascading failure process, which helps to understand the mechanism of failure propagation in the network system. The model describes a network composed of identical components with load distributed uniformly in [*L*_min_, *L*_max_], and the average initial component loading *L* = (*L*_min_+*L*_max_)/2. An initial disturbance *D* is added to all components, and may cause some components to exceed their capacity threshold *L*_fail_ = 1, which is assumed identical for all the components. If component *j* is working and *L_j_*+*D>L*_fail_, component *j* fails. Then, each failure of a component leads to an additional load *P*>0 added to all the other functional components in the network, which may cause further failures in a cascade.

The restoration actions will be considered once the cascading failures process has been triggered. A typical in-process restoration procedure is comprised of three stages [33], [34]: firstly, estimating system/component status, locating the critical loads, and developing the strategies for rebuilding the network connections; secondly, identifying the paths of restoration, energizing and interconnecting subsystems; thirdly, restoring most of lost loads. Restoration strategies differ from each other in the above aspects. Here we propose a restoration model considering the timing and strength of restoration, which mainly determine the effects of restoration. In the model, each failed component is repaired with a certain probability *p_r_* at a given step *t_r_*>0 during cascading failure. The restoration actions recover the links of the component to be repaired, while its links to failed components remain disconnected. We assume that restoration may cause some disturbance to the existing functional components in the network. We model this restoration disturbance by adding a random perturbation *D_r_* distributed uniformly in 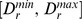
to the load of each functional component. The value of restoration disturbance depends on whether the restoration action is implemented appropriately to the system, which could be positive or negative. This means that the restoration may either reduce or increase the loads of the functional components, depending on whether it is beneficial or harmful.

The following algorithm is used to realize the above procedure. The details of the algorithm are summarized as follows:

1. All *N* components are initially functional and loaded by quantities *L_1_*, *L_2_*, …, *L_n_*, which are independent random variables uniformly distributed in [*L*_min_, *L*_max_]. Initialize the stage counter *t* to zero.
2. Add the initial disturbance load *D* to the load of each component: then, the load of component *j* is *L_j_*+*D*.
3. Each existing component is examined: if the current load of component *j* is larger than *L_fail_*, component *j* fails. We denote the number of components failed in this single step by *M_t_*. Add *M_t_P* to the load of each functional component. The stage counter *t* is incremented by one.
4. When *t* reaches the restoration moment *tr*, i.e., *t* = *tr*, each failed component is repaired with probability *pr* by reconnecting it to its adjacent functional components. The load of each repaired component is reassigned uniformly in [*L*_min_, *L*_max_]. Add a random disturbance *Dr* uniformly distributed in 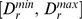 to the load of each functional component.
5. Go back to step 3, unless the cascading failures stop.

## Effects of Restoration Strategies

**Figure 1:**
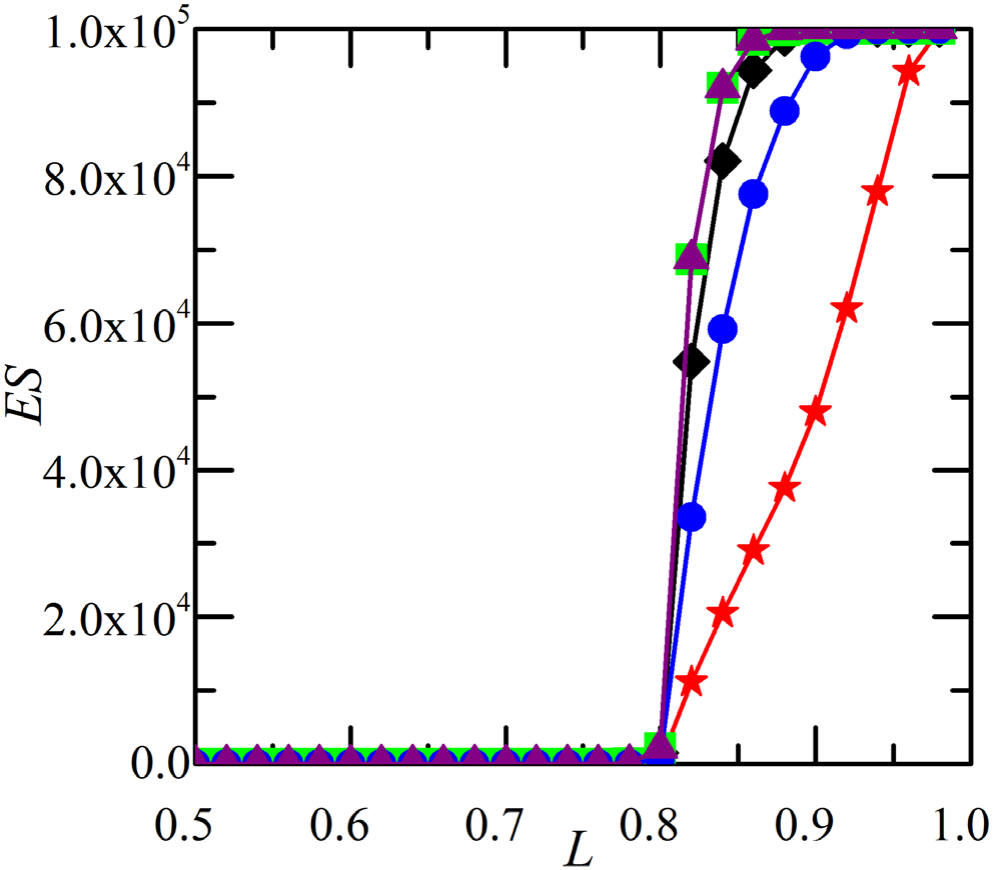
Average avalanche size *ES* as a function of the system loading level *L*. Results for five different restoration strategies: (1) *p_r_* = 0 (black diamonds); (2) *t_r_* = 1, *p_r_* = 1, 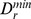 = 6×10^−6^, 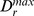 = 8×10^−6^ (green squares); (3) *t_r_* = 4, *p_r_* = 1, 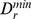 =  6×10^−6^, 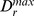 = 8×10^−6^ (purple triangles); (4) *t_r_* = 1, *p_r_* = 1, 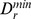 = −8×10^−6^, 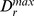 = −6×10^−6^ (red stars); (5) *t_r_* = 4, *p_r_* = 1, 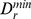 = −8×10^−6^, 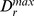 = −6×10^−6^ (blue circles). Each curve corresponds to the average over twenty thousand realizations of networks with 10^5^ components. The example network system has no specific topology, on which the results do not depend. The initial component loading can vary from *L*_min_ to *L*_max_ = *L*_fail_ = 1. Then, *L* = (*L*_min_+1)/2 may be increased by increasing *L*_min_. The initial disturbance *D  = * 4×10^−6^ is assumed to be the same as the load transfer amount *P = *4×10^−6^. All the investigated network systems without restoration satisfy the cascading condition that the cascade step is no less than 5.

In this section, we study the effects of different restoration strategies on the system robustness against cascading failures and the resulting system reliability. We begin our study by evaluating the restoration effect on the total damage made by cascading failure (the average avalanche size). Figure 1 compares different restoration strategies in terms of restoration timing *t_r_* and strength *p_r_* by measuring the number of failed components *ES*. As shown in [44], there is a transition of *ES* occurring at critical point *L*_c_ = 0.8 without restoration (*p_r_* = 0). When the value of *L* is below this threshold, few failures emerge. On the other hand, for *L* above the threshold, there is a significant risk of cascading failures that lead to global collapse of the system. And in-process restoration can reduce the final damage significantly if it is implemented properly. As shown in Fig. 1, cascading failure under restoration (*p_r_*>0) with negative *D_r_* generates much smaller avalanche size *ES* than the case without restoration (*p_r_* = 0). Furthermore, for negative *D_r_*, early restoration (e.g., *t_r_* = 1) ends up with more functional components than late restoration (e.g., *t_r_* = 4). For positive *D_r_*, restoration worsens the system in terms of *ES*.

To investigate the effects of different restoration strategies on improving the reliability against cascading failures, we measure the system load fluctuations (*SLF*) defined as
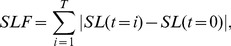
(1)where
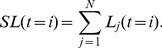
(2)
*N* is the total number of the components in the network system. *L_j_* is the load of component *j*, and we set *L_j_* (*t* = *i*)  = 0 if component *j* is failed at the moment *i*. *SL*(*t* = 0) is the initial system load when the system maintains its normal functional state. *SL*(*t* = *i*) is the total load of system at the moment *i* in the cascading process, i.e., the sum of the loads of all functional components. The parameter *T* is the duration of the whole dynamical process of cascading failures.

**Figure 2:**
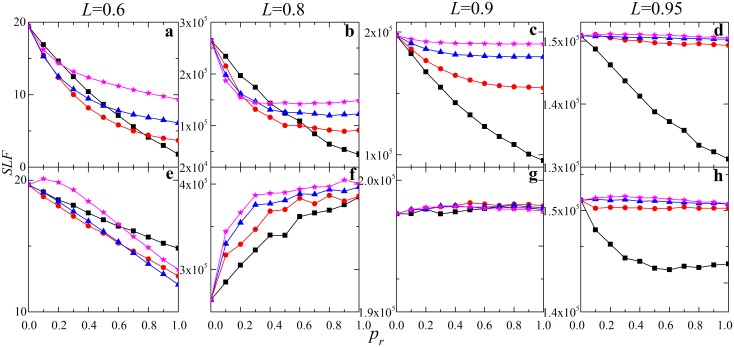
System load fluctuations *SLF* as a function of restoration probability *p_r_* for different system loading level *L*. According to the proposed modeling framework for restoration, we compare different strategies in four cases of *t_r_* = 1 (black squares), *t_r_* = 2 (red circles), *t_r_* = 3 (blue triangles), *t_r_* = 4 (magenta stars). 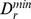 = −8×10^−6^, 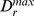 = −6×10^−6^ for the panel above, and 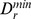 = 6×10^−6^, 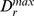 = 8×10^−6^ for the panel below. The other parameters are the same as in Fig. 1.

The measure *SLF* reflects the system instability in the whole process of cascading failures, considering the required balance between the supply and demand. Figure 2 shows *SLF* under restoration at a given restoration timing *t_r_* as a function of the restoration probability *p_r_*. From Figs. 2a–2d, we can see that the restoration with negative disturbance can effectively mitigate cascading failures and reduce system instability. Furthermore, the system can be improved by high strength of restoration.

The situation for positive restoration disturbance is more surprising. One may expect that restoration would worsen the cascading failure when *D_r_* is positive. The corresponding results with positive *D_r_* are complicated: restoration can still improve the system for subcritical loading (Fig. 2e); at critical loading *L_c_*, restoration produces quite large *SLF* and induces extra instability (Fig. 2f); for supercritical loading, restoration has almost no impact on *SLF* (e.g., *L* = 0.9, Fig. 2g).

The results above can be explained as follows. The restoration effect is dominated by two factors, restored components and the consequential restoration disturbance. These two factors are cooperative under negative *D_r_* so that failed components are recovered, when the load of functional components is decreased. This cooperative effect under negative *D_r_* can be stronger for early restoration. When *D_r_* is positive, however, restoration will increase the load of functional components when failed components are restored at the same time. The outcome of restoration then depends on the competition between these two factors.

**Figure 3:**
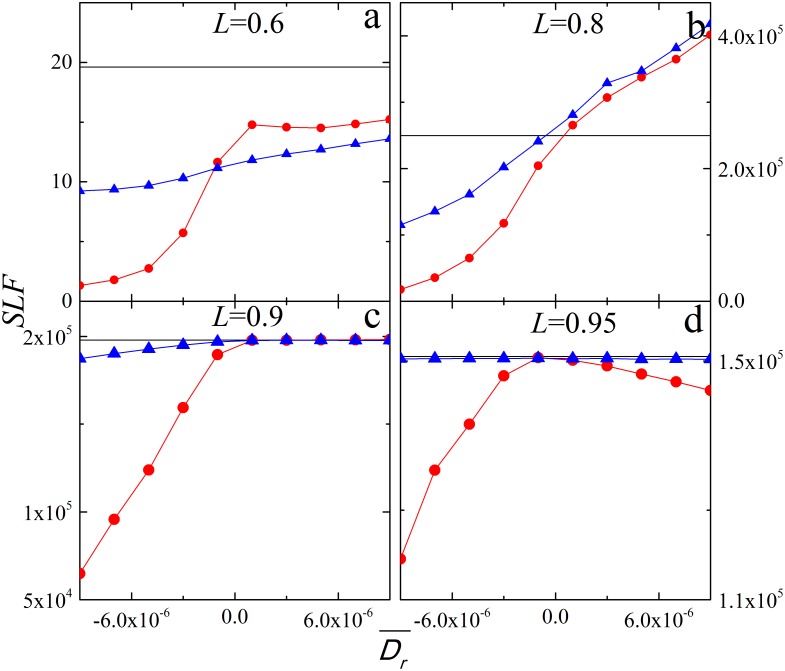
System load fluctuations *SLF* as a function of the average restoration disturbance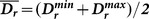 for different system loading level *L*. Results for different restoration strategies: (1) *p_r_* = 0 (black, straight line); (2) *t_r_* = 1, *p_r_* = 1 (red circles); (3) *t_r_* = 4, *p_r_* = 1 (blue triangles). Here we set 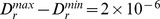. The other parameters are the same as in Fig. 1. Notice that *SLF* in case of *p_r_* = 0 remains constant as *SLF* is independent of *D_r_* without restoration.

To further explore the effect of restoration disturbance, in Fig. 3 we analyze the restoration effect as a function of the restoration disturbance. For *L* = 0.6 and 0.8, restoration (*p_r_* = 1) significantly increases *SLF* as the restoration disturbance increases (Figs. 3a and 3b). For supercritical loading, *SLF* increases for negative *D_r_* and then remains saturated for positive *D_r_* (*L* = 0.9, Fig. 3c), while early restoration (*t_r_* = 1) can improve system for both negative and positive *D_r_* (*L* = 0.95, Fig. 3d). Similar as the results in Fig. 2, restoration under negative *D_r_* at an early cascade step is beneficial for all investigated cases. When restoration disturbance *D_r_* is positive, restoration improves system only for certain values of system loading.

To observe the dynamical processes of restoration, we track the system evolution under restoration in terms of system fluctuations during cascading failure. The load fluctuation of the system at the moment *t* is defined as
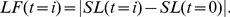
(3)


**Figure 4:**
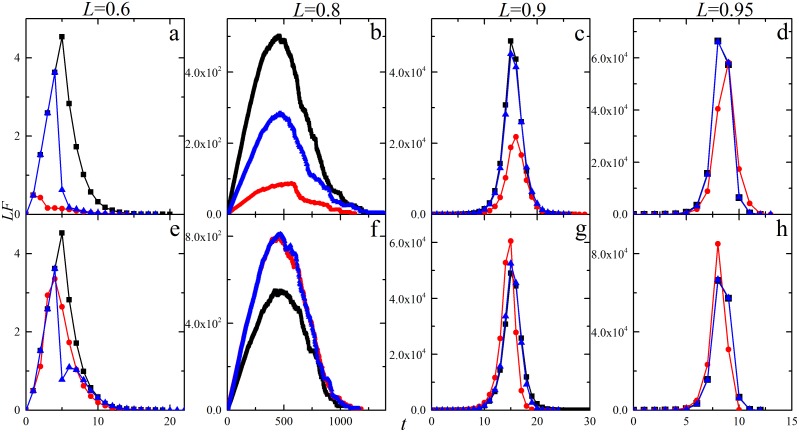
Load fluctuations *LF*(*t*) during cascading failures. Results for different restoration strategies: *p_r_* = 0 (black squares), *t_r_* = 1, *p_r_* = 1 (red circles) and *t_r_* = 4, *p_r_* = 1 (blue triangles). Here, the *x* axis is the system unstable moment *t* based on cascade and restoration. 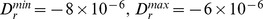 for the panel above and 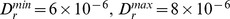 for the panel below. The other parameters are the same as in Fig. 1.

For convenience, here we assume that *LF*(*t*) = 0 when *t*>*T.* Figure 4 demonstrates the system evolution process in terms of load fluctuation *LF*(*t*), where total system load fluctuation is the corresponding area under the curve of *LF*(*t*). Early restoration (*t_r_* = 1) under negative *D_r_* is shown to reduce the load fluctuation since the restoration moment in the process of cascading failures (Figs. 4a–4d). However, for positive *D_r_*, load fluctuation of restoration at *L* = 0.6 is lower than that without restoration (Fig. 4e), while for *L* = 0.8 load fluctuation is significantly increased (Fig. 4f). And it is not helpful to restore system late with positive *D_r_* for a system high loaded (Figs. 4g and 4h).

## Analytical Methods

According to the proposed restoration model, *n* components are loaded in [*L*_min_, *L*_max_]. We set  = (*L*_min_+*L*_max_)/2 and *L*_max_ = *L*_fail_ = 1. Then component *j* has the load *L_j_*∈[2*L*-1, 1] and fails when its load is larger than *L*_fail_. An initial disturbance *D* is added to each component. Each failed component transfers a fixed amount of load *P* to other functional components.

Based on the literature [44], the distribution of the total number of failed components *S* without restoration can be given by
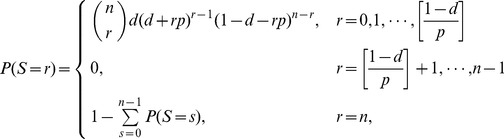
(4)where, [*x*] is the largest integer not more than *x* and 
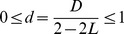
, 
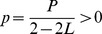
, 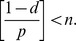


When 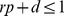, 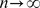, 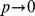, 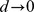, 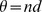, 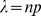, the above distribution can be approximated by a branching process with
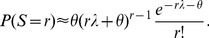
(5)


Then we have the approximation [45] based on the property of this branching process
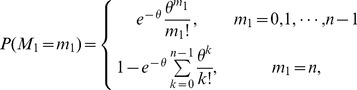
(6)


and
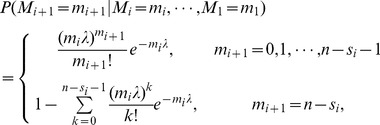
(7)


Where 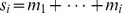.

As our investigated configurations satisfy the cascading condition that the cascade step is no less than 5 (or any arbitrary number), we obtain 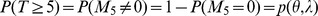. Then the distribution of the total number of failed components *S* without restoration (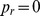) is
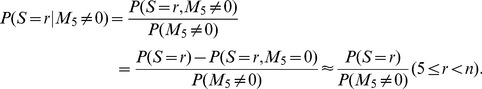
(8)


According the parameters in the text, we set 
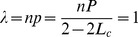
 and get the critical loading 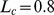, which corresponds to the case in Fig. 1.

When the restoration strategy (*t_r_*, *p_r_*) (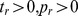) is taken, we assume that the total number of components failed at restoration timing is 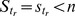. Then the current state of the system is as follows: *m* failed components, 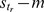 restored components loaded in 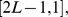 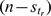 functional components loaded in 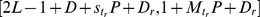, and the failed rate is 
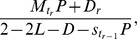
 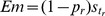. Then the system may go on evolving after the restoration. And we can clearly know the average avalanche size *ES* is strongly dependent on the value and sign symbol of the restoration disturbance 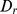, the restoration timing *t_r_*, the restoration strength *p_r_* and the system loading level *L*.

When 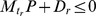, restoration ends the cascading failure. Then the distribution of the total number of components failed *S* with restoration is
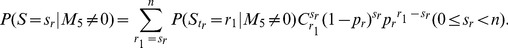
(9)


When 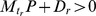, the state of system at *t_r_* can be replaced by *m* failed components and (*n*−*m*) functional components loaded in 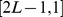 disturbed by the load 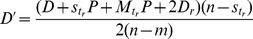. Considering the cascading condition that the cascade step is no less than 5 (or any arbitrary number) and the restoration timing *t_r_*, the distribution of the total number of components failed *S* with restoration is
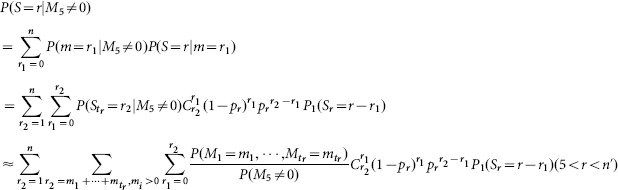
(10)


And the distribution of the total number of components failed *S_r_* after restoration is
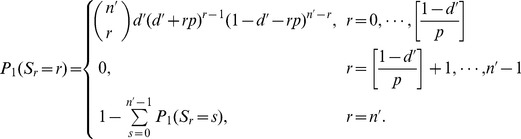
(11)where 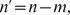 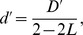 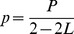.

**Figure 5:**
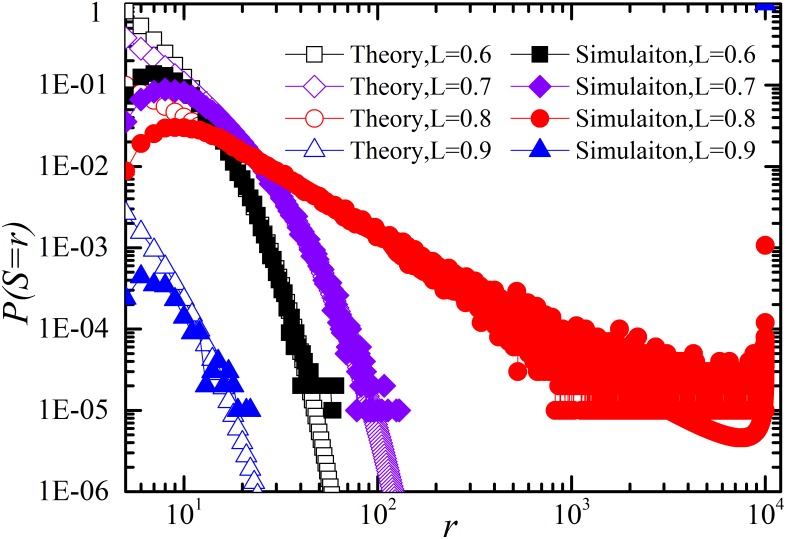
Log-log plot of distribution of number of components failed *S* for different system loading level *L* without restoration (*p_r_ = *0). Note the power-law region for the critical loading *L* = 0.8. Simulation results are averaged over 100,000 realizations of the systems. The related parameters are *N* = 10000, *D* = *P* = 0.00004. Note that the simulation results coincide well with theoretic analysis.

Next we give the analytical results for the proposed modeling framework of restoration. Firstly, we give the comparison between the simulation and theory in case of *p_r_* = 0 in Fig. 5. The case corresponds to Eq. (8). As shown in Fig. 5, theoretical calculation coincides well with the numerical simulations. And the distribution behaves as a power-law at the critical loading, at which system has a high probability of large-scale failures.

**Figure 6:**
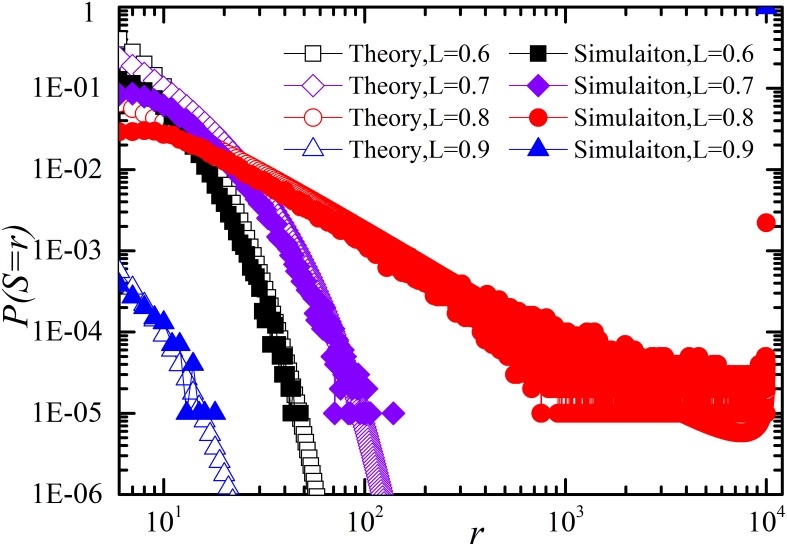
Log-log plot of distribution of number of components failed *S* for different system loading level *L* with restoration (*t_r_* = 1, *p_r_ = *1). 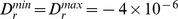
 and the other parameters are the same as in Fig. 5. Note that the simulation results coincide well with theoretic analysis.

**Figure 7:**
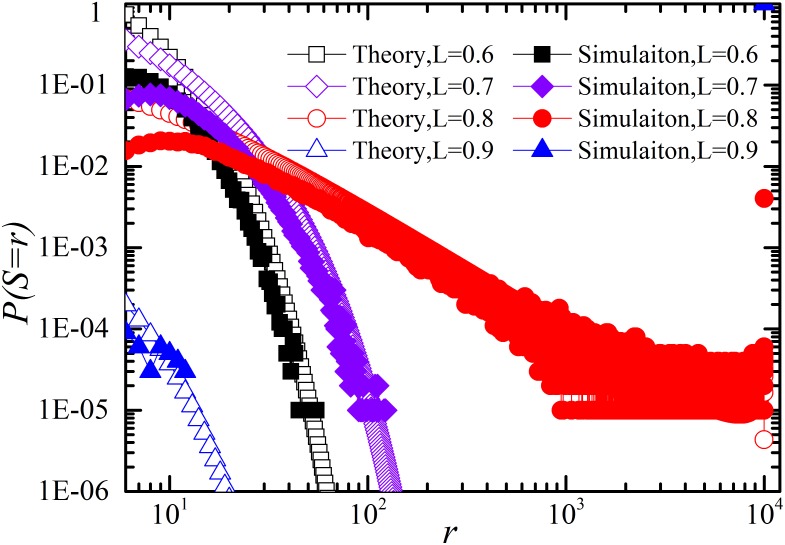
Log-log plot of distribution of number of components failed *S* for different system loading level *L* with restoration (*t_r_* = 1, *p_r_ = *1). 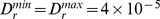
 and the other parameters are the same as in Fig. 5. Note that the simulation results coincide well with theoretic analysis.

Then, we give the comparison of restoration between simulation and theory in Fig. 6 for negative *D_r_* and Fig. 7 for positive *D_r_* in case of *p_r_*≠0. These cases correspond to Eq. (10). As shown in Fig. 6 and Fig. 7, theoretical calculation coincides well with the numerical simulations.

## Model Variations

**Figure 8:**
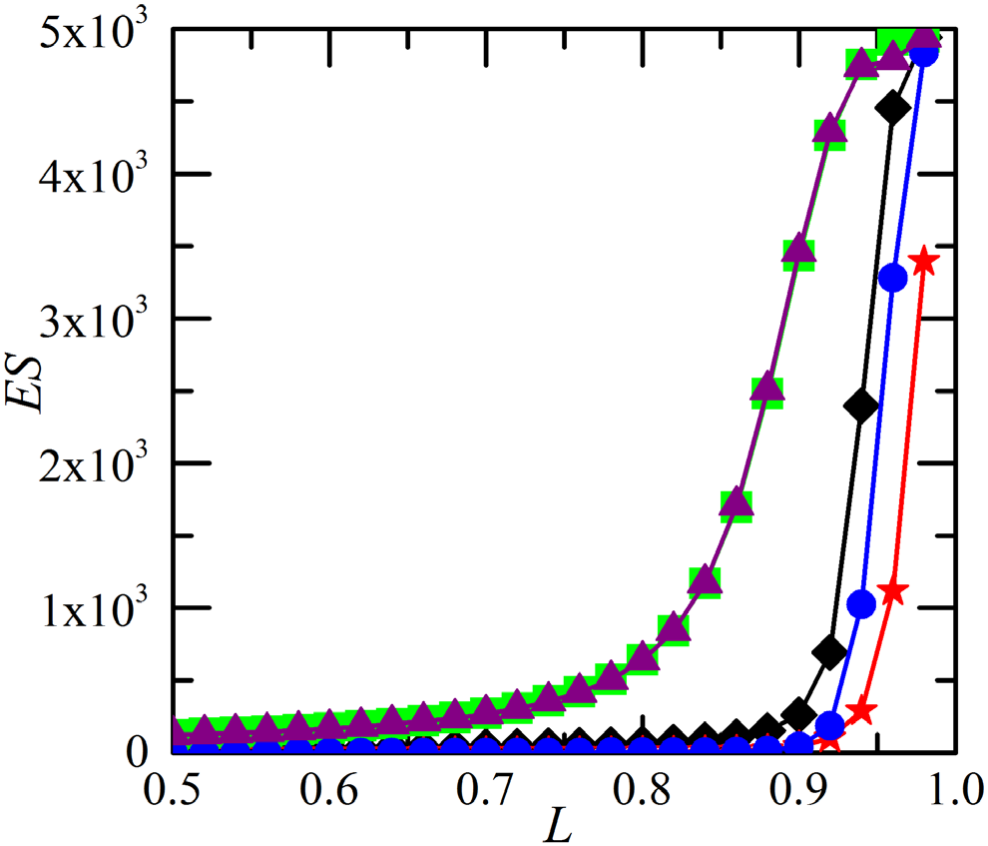
Average avalanche size *ES* as a function of the system loading level *L* in power grid. Results for five different restoration strategies: (1) *p_r_* = 0 (black diamonds); (2) *t_r_* = 1, *p_r_* = 1, 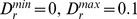 (green squares); (3) *t_r_* = 4, *p_r_* = 1, 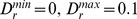 (purple triangles); (4) *t_r_* = 1, *p_r_* = 1, 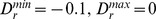 (red stars); (5) *t_r_* = 4, *p_r_* = 1, 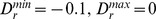 (blue circles). Results are averaged over 1,000 realizations. All components are initially loaded by independent random variables *L_1_*, *L_2_*, …, *L_n_* from Gaussian distribution 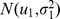 in [*L*_min_, *L*_max_], and *D_r_* follows Gaussian distribution 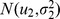 in 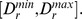 The model parameters are the same in all simulations: *L*_max_ = *L*_fail_ = 1, 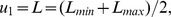 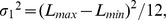 *D = *0.01, *Q = *0.05, 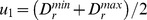 and 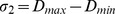.

**Figure 9:**
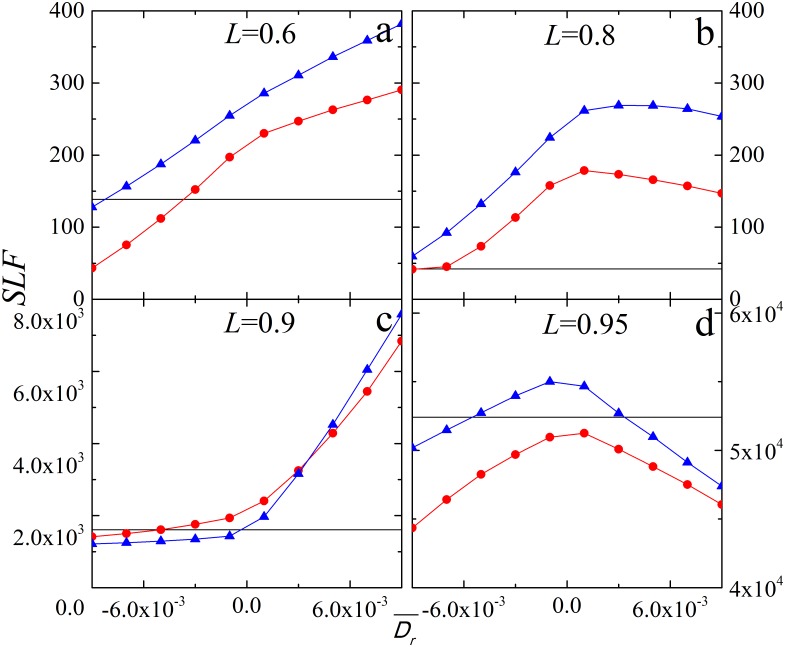
System load fluctuations *SLF* as a function of the average restoration disturbance 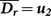 for different system loading level *L* in power grid. Results for different restoration strategies: (1) *p_r_* = 0 (black, no symbols); (2) *t_r_* = 1, *p_r_* = 1 (red circles); (3) *t_r_* = 4, *p_r_* = 1 (blue triangles). Here we set 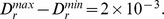 The other parameters are the same as in Fig. 8.

We apply our modeling framework of restoration to the western U.S. power transmission grid [46] for the model validation. Here we present the results in Fig. 8 and Fig. 9 on the realistic power system with more practical consideration in the model:

Variation 1: initial load distribution. We change the distribution of initial component loading from uniform distribution to Gaussian distribution;

Variation 2: impact of each failed component on the functional components. Previously, each failure of a component leads to an additional load *P*>0 added to all the other functional components in the network regardless of network topology. Now each failed component leads to an additional load *Q*>0 only added to its functional neighbors, which is dependent on network topology;

Variation 3: restoration disturbance *D_r_*. We change the distribution of restoration disturbance from uniform distribution to Gaussian distribution.

Figure 8 compares different restoration strategies in terms of restoration timing *t_r_* and strength *p_r_* by measuring *ES*. There is a transition of *ES* occurring around critical point *L*_c_ = 0.9 without restoration (*p_r_* = 0). As shown in Fig. 8, cascading failure under restoration (*p_r_*>0) with negative *D_r_* generates smaller *ES* than the case without restoration (*p_r_* = 0). For negative *D_r_*, early restoration (e.g., *t_r_* = 1) ends up with more functional components than late restoration (e.g., *t_r_* = 4). For positive *D_r_*, restoration worsens the system in terms of *ES*. The results are similar to Fig. 1.

Figure 9 further explores the effect of restoration disturbance in terms of the system load fluctuations (*SLF*). We can see the effects of restoration are heavily influenced by the restoration strategies. For subcritical loading (*L* = 0.8), *SLF* increases for negative *D_r_* and almost stays constant for positive *D_r_*, while restoration will worsen system for each *D_r_* (Fig. 9b). For supercritical loading (*L* = 0.95), *SLF* increases for negative *D_r_* and decreases for positive *D_r_*, while early restoration (*t_r_* = 1) will improve system for each *D_r_* (Fig. 9d). Restoration can improve system only for certain values of system loading for a given *D_r_*.

## Conclusions

Proper restoration during cascading failures can actively prevent failure propagation through the entire network. We have proposed a novel modeling framework to investigate restoration effect during cascading failures with respect to restoration timing *t_r_* and strength *p_r_*. The model also considers additional disturbances on the system due to the restoration actions themselves. The effects of the restoration have been analyzed with respect to the mean number of failed components *ES* and the system load fluctuations *SLF*. *ES* focuses on the final state of the cascade-restoration process, whereas the newly introduced measure *SLF* describes the dynamical behavior of the systems.

By applying the proposed modeling framework on the example system, we find that the restoration effects also depend on the combination of system loading level *L* and restoration disturbance *D_r_*. Although the system can be improved by proper in-process restoration, the application of restoration should be implemented carefully considering the system loading level. Our framework and findings can help to evaluate restoration scheme of complex systems and provide insights into the development of optimal restoration strategy against cascading failures, which are helpful for guiding improvements of reliability and robustness of actual network systems.

Given the rapid development of Micro-Grid technology, it is interesting and necessary to study the restoration for Micro-Grid against cascading failures. Although, for now we have no data for the Micro-Grid, we will perform the relevant study in the future based on the framework provided in this paper. Based on our framework provided in the paper, more realistic scenario considering system real-time status can also be studied in the near future.
